# Expanding the toolkit: a call for embracing methodological diversity in healthcare professions education research

**DOI:** 10.3389/fmed.2025.1712612

**Published:** 2025-12-08

**Authors:** Louise M. Allen, Lynn V. Monrouxe

**Affiliations:** 1 Department of Medical Education, The University of Melbourne, Melbourne, VIC, Australia; 2 Faculty of Medicine Nursing and Health Sciences, Monash University, Clayton, VIC, Australia; 3 Faculty of Medicine and Health, The University of Sydney, Sydney, NSW, Australia

**Keywords:** research methods, health professions education, methodological diversity, research capacity building, research ecosystem

## 1 Introduction

Abraham Maslow once cautioned: “If the only tool you have is a hammer, everything looks like a nail.” In healthcare professions education research, this warning carries weight. Despite our field's impressive growth—submissions to Frontiers in Medicine, Healthcare Professions Education have risen from 90 in 2021 to around 500 in 2025, echoing the general rise in increasing journal submissions in our field—we face an uncomfortable truth: our methodological toolkit hasn't expanded at the same pace as our scholarly output. While the range of methods being used by the HPE community is growing, there is an over reliance on a smaller number of approaches (1, 2). For example, a recent review that examined trends in healthcare professions education research in 2000, 2010 and 2020 showed that over the last two decades there has a been a gradual increase in the use of qualitative methodologies and little change in the use of mix methods, however quantitative methodologies are still predominant (1). While studies using quantitative methodologies provide important insights for our field they have limitations in answering how and why questions and thus they are not best positioned to gain understanding of the complex, contextual, and socially mediated problems we face in healthcare professions education. Beyond this, when qualitative methodologies are used approximately 2/3 of the papers either don't specify a specific qualitative design or use grounded theory or qualitative description (1). There is limited use of study designs such as discourse analysis and participatory approaches that can provide rich insights into underrepresented voices in healthcare professions education, and designs such as realist evaluation which provide rich insights into how and why questions.

This matters more than we might initially think. There are multiple papers published in the last year that are either encouraging the increased use of more diverse methods (3–5), or demonstrating a need for more diversity in the methods we use (1, 2). The apparent increase in publication of methodology papers is positive. These papers can help us to embrace new research tools, making people aware of these approaches and providing insights into how to use them. But the overreliance on a limited set of research methods is problematic because we face increasingly complex problems requiring the use of multiple tools at our disposal. Why do we need to use multiple tools? If as a field we rely too heavily on a small range of research methods and theoretical perspectives then we risk illuminating only a limited perspective of a topic. Different methodological traditions embody different epistemological assumptions about knowledge and truth, and over-reliance on any single approach risks impoverishing our understanding of complex educational phenomena that require multiple ways of knowing to fully comprehend. Embracing more innovative and diverse methodologies and methods allows us to examine problems from different perspectives, providing varied insights, enhancing the breadth and depth of understanding for a fuller and more nuanced picture.

For example, the widely accepted notion of empathy decline (6) in medical training exemplifies how over-reliance on research methods (e.g., the Jefferson Scale of Empathy or the Balanced Emotional Empathy Scale) can lead to potentially misleading conclusions. Indeed, this narrative faces pushback from studies using different methods. Take Monrouxe et al.'s large-scale UK survey (7), which examined healthcare students' responses to moral distress resulting from experiences of professionalism conflicts. A strikingly different picture is found: rather than becoming numb to poor treatment of patients, students actually become more distressed when they witness unprofessional behavior toward those in their care in situations not essential for learning. This contradicts the idea that medical training inevitably erodes empathy. Instead, it highlights a crucial point about our research: the lens through which we examine questions—our methods and theoretical frameworks—fundamentally shapes what we discover. However, we shouldn't simply abandon current methods that are commonly used. To balance maintaining our field's existing expertise with continuing to grow and diversify it, we propose considering multiple levels of our research system: individual researchers and research teams, research training organizations, and journals.

## 2 Discussion

### 2.1 Individual researchers and research teams

Our research conversations include contributions from dedicated healthcare professions education researchers, education or teaching specialists, clinician educators, administrators, consumers, and students (to name a few). Each has different capacity to develop expertise in new methods and theories. For those with limited time, the barrier to acquiring expertise in new methods may seem insurmountable, highlighting the importance of including methodological experts in research teams when exploring unfamiliar approaches. For those with more dedicated time for research, it is important to actively broaden their skillset. However, identifying what we don't know, or the next logical step in our learning, can be challenging. Avoiding solely upskilling in familiar methods and theories is key. Instead, we advocate for pushing beyond your comfort zone, deliberately seeking opportunities to expand expertise incrementally, focusing on approaches adjacent to your current skillset that offer new perspectives or insights. For example, a qualitative researcher trained in constructivist grounded theory and narrative approaches from an interpretivist perspective has always positioned learners and educators as research subjects rather than partners. After being introduced to participatory approaches by a colleague, they attend a workshop on co-design principles and read literature on student-as-partner initiatives and critical inquiry. The researcher recognizes that traditional researcher-led approaches miss crucial insider knowledge and perpetuate power imbalances. The researcher plans to include learners and educators as co-investigators on their next grant submission, as well as their colleague who has methodological expertise in participatory approaches. Some strategies to assist with expanding expertise incrementally include engaging with experts in unfamiliar methods, seeking mentorship, practicing reflection (particularly on epistemic views and potential paradigm shifts), exploring broader literature or conference presentations, and leveraging AI tools like MedEd Mentor.

### 2.2 Research training organizations

#### 2.2.1 Leaders of short courses and degrees in healthcare professions education

The number of short courses and degrees in healthcare professions education and associated research is growing rapidly (8, 9). These courses develop future healthcare professions education leaders. Therefore, educators designing and evaluating these courses must examine the research methods and theoretical frameworks they include, ensuring a diverse and comprehensive methodological foundation with an view to understanding different ways of knowing (10). Courses could incorporate modules on increasingly recognized research approaches such as Indigenous research methodologies, participatory and co-design approaches, and longitudinal qualitative research alongside traditional methods, integrating case studies demonstrating methodological diversity's value in addressing complex healthcare professions education questions (10).

#### 2.2.2 Graduate research supervisors

Furthermore, supervisors of graduate research students and early career researchers can foster methodological breadth by actively engaging in conversations about exploring diverse methods and theories, encouraging consideration how different approaches might yield novel insights into their research questions. For example, a supervisor might ask a student planning a survey on clinical reasoning: What would we learn if we observed learners thinking aloud during a complex case instead or interviewed them about their decision-making process? How might those approaches reveal reasoning patterns that questionnaires cannot?

### 2.3 Journals

Editors, deputy editors, and reviewers shape the landscape of our published research, as such they each hold positions of power to influence the field.

#### 2.3.1 Reviewer-level actions

Reviewers should embrace new methodological approaches, recognizing that novel methods offer fresh perspectives on longstanding issues. Rather than dismissing unfamiliar designs as lacking rigor, reviewers are encouraged to read up on novel methods, and they can evaluate methodological fit by asking questions such as: Does the method align with the research question and philosophical positioning? What quality reporting standards/guidelines are appropriate to assess the quality of the study? Is there sufficient methodological detail included to make a judgement on the quality of the study? For example, a narrative inquiry study examining learner experiences of burnout should not be judged against RCT criteria; instead, reviewers might assess trustworthiness, reflexivity, and transferability (11). By adopting this approach reviewers signal to the field that diverse approaches strengthen healthcare professions education research.

#### 2.3.2 Editor-level actions

Editors and deputy editors can actively advocate for methodological diversity through strategic initiatives. They can introduce and promote article types dedicated to methodological exploration, such as Frontier in Medicine's “Methods” article type and Medical Education's “Focus on Research Methods” and “Cross Cutting Edge” sections. These platforms showcase innovative approaches, encouraging researchers to submit papers that push methodological boundaries. Furthermore, editors can demonstrate openness to new methodologies and theoretical perspectives in their editorial decisions, signalling that diverse approaches are valued. These dedicated methodological platforms serve multiple functions: they provide a legitimized space for methodological innovation, creating what Wenger might call a “community of practice” around diverse approaches. They also reduce the risk for authors who might otherwise hesitate to submit methodologically novel work, uncertain whether their approach will be valued. By institutionalizing methodological diversity through specific article types, journals signal that innovation in “how we know” is as valued as innovation in “what we know”: a message that can reshape submission patterns and normalize methodological experimentation.

#### 2.3.3 Journal-level strategies

Finally, editors could use special issues or themed editions to curate articles examining specific topics through various methodological lenses. While systematic evidence of special issues' effectiveness in promoting methodological diversity remains limited, we can draw on broader publishing scholarship and our own field's experience. Thematic collections create what Kuhn described as moments of “paradigmatic visibility”: making alternative approaches tangible rather than abstract. Medical Education's “Cross Cutting Edge” series, for instance, demonstrates how curated collections can showcase a single phenomenon examined through multiple methodological lenses, making the value of diversity experientially clear to readers. Moreover, when accompanied by thoughtful editorials that explicitly compare and contrast what different methods revealed, these collections serve a pedagogical function: teaching the field about epistemic gains from methodological pluralism itself.

### 2.4 Conclusion

In conclusion, embracing methodological diversity in healthcare professions education is not, and should not be an individual endeavor. Maslow's hammer analogy reminds us that our methodological choices fundamentally shape what we can discover—yet this constraint operates not only at the individual researcher level but also systemically. The path forward requires coordinated action across all levels of our research ecosystem, from individual researchers pushing their methodological boundaries to research training organizations and journals actively promoting diverse approaches. Only through such comprehensive efforts can we ensure that our growing field develops the nuanced understanding necessary to prepare healthcare professionals for an increasingly complex world. By expanding our toolkit and embracing methodological diversity—whilst maintaining our hard-earned expertise—we position ourselves to better understand and address the complex challenges facing our field.
